# Social Efficiency of Public Transportation Policy in Response to COVID-19: Model Development and Application to Intercity Buses in Seoul Metropolitan Area

**DOI:** 10.3390/ijerph191912060

**Published:** 2022-09-23

**Authors:** Junsik Park, Gurjoong Kim

**Affiliations:** Korea Transport Institute, 370 Sicheong-daero, Sejong-si 30147, Korea

**Keywords:** COVID-19, public transportation, passenger reduction policy, intercity bus, metropolitan area, social efficiency

## Abstract

Although more than two years have passed since the appearance of the coronavirus disease 2019 (COVID-19), few policies on public transportation have been implemented to reduce its spread. It is common knowledge that public transportation is vulnerable to COVID-19, but it has not been easy to formulate an appropriate public transportation policy based on a valid rationale. In this study, a modified SEIHR model was developed to evaluate the socioeconomic effects of public transportation policies. By applying the developed model to intercity buses in the Seoul metropolitan area, the socioeconomic efficiency of the policy of reducing the number of passengers was evaluated. The analysis showed that the optimal number of passengers decreased as the number of initially infected people increased; in addition, the basic reproduction number *R_0_*, illness cost per person, and probability of infection with a single virus were higher. However, depending on these variable conditions, the policy to reduce the number of passengers in a vehicle may not be required, so it is necessary to make an appropriate judgment according to the situation. In particular, the emergence of a new mutant COVID-19 will necessitate the development of appropriate countermeasures by comprehensively examining the change in the number of infected individuals and the fatality rate. This study can guide the development of such countermeasures.

## 1. Introduction

After the outbreak of the coronavirus disease 2019 (COVID-19), China locked down the Wuhan area, Australia and New Zealand closed their borders, and some European countries closed commercial outlets and restricted the movement of people. Nevertheless, the disease has spread rapidly to all countries. COVID-19 was expected to end after a vaccine was developed. However, despite the fact that the majority of the population has been vaccinated, COVID-19 continues to spread. Repeated increases and decreases in proliferation continue to occur, and no country has been able to stop the spread of COVID-19. However, depending on the response method, the timing and degree of spread of COVID-19 differ, resulting in differences in the ratio of the number of severe cases and deaths to the number of confirmed cases.

In countries where the number of infections increased rapidly over a short period of time, inadequate medical facilities resulted in a lack of appropriate treatment for the infected, leading to numerous deaths. Once a large portion of the population was infected and collective immunity was attained, the quarantine policy was abolished. In the absence of clear quarantine measures, COVID-19 swept through countries like a tsunami.

Korea has fewer confirmed cases than other countries, even though it has not implemented a regional lockdown or restricted public movement. Since the outbreak of the omicron mutant of COVID-19, the number of confirmed cases has increased rapidly; however, due to the high vaccination rate, the number of severe cases and deaths has not increased significantly, and the medical system has not collapsed.

Many experts worldwide have acknowledged the excellence of Korea’s quarantine policy. Preemptive and exemplary measures to prevent the spread of infection, such as personal hygiene measures, including wearing a mask, systematic and thorough epidemiological investigation, pre-isolation measures for contacts, social distancing policies, and high vaccination rates, were systematically implemented. People cooperated with the government and abided by the preventive measures. 

As of August 2022, although the rising trend of the spread of the omicron mutant of COVID-19 appears to have stopped to a certain extent, more than 100,000 confirmed cases continue to be recorded every day, and the duration of the pandemic remains unknown [1]. Therefore, additional measures are required to reduce the spread of COVID-19 in Korea as well as other countries.

Public transportation has a high likelihood of spreading COVID-19 because large numbers of commuters are confined in small spaces. Therefore, although public transportation is sometimes recognized as a major factor in the spread of COVID-19, a public transportation policy to prevent the spread of COVID-19 infection has rarely been implemented. This may be because such a policy is difficult to implement in reality. Further, it is impossible to determine the social efficiency of the policy because the contribution of public transportation to the spread of COVID-19 infection as well as the extent to which the spread of COVID-19 infection can be limited by implementing appropriate transportation policies is not known. Therefore, it is necessary to develop a public transportation policy that is based on a valid rationale to reduce the spread of COVID-19.

Few studies have been conducted on public transportation policies to respond to COVID-19 or to assess the probability of infection owing to the use of public transportation. Kamga and Eickemeyer [2] reviewed public transport policies in response to COVID-19, implemented in the United States and Canada. According to the study, 6-foot (1.8 m) distancing was implemented in stations, trains, subways, and bus vehicles in several states, and operating plans were changed, for example, additional trains were deployed to support the distancing policy. These public transport policies have generally been claimed to be effective in curbing the spread of COVID-19.

The Rail Safety and Standards Board [3] estimated the probability of COVID-19 infection in railroads by considering the probability of encountering an infected person in a railroad car and the probability of transmitting the infection. There is a limit to estimating the probability of infection according to specific changes in the situation because preset values are applied rather than directly estimating the probability of encountering an infected person and the probability of acquiring the infection.

Recently, Park and Kim [4] developed a model to estimate the risk of COVID-19 infection on public transportation based on the model proposed by Lelieveld et al. [5] and assessed the effectiveness of the measures in reducing the risk of infection. Lelieveld et al. [5] estimated the probability of infection by assuming that the virus is uniformly distributed in a general indoor space, whereas Park and Kim [4] estimated the probability of infection by developing a model that can consider two cases, namely, the virus is uniformly distributed in a bus and the probability of infection decreases with distance. It was found that in addition to wearing a mask and refraining from talking in public transportation vehicles, ventilation, distributed seating arrangement, and reduction in the number of passengers were effective in lowering the probability of COVID-19 infection. 

In this study, a modified susceptible–infected–recovered (SIR) model, which is a representative infection transmission model, was used to estimate the social cost of COVID-19. The social cost is defined as the sum of the cost incurred by promoting policy for the management of infectious disease and the illness cost incurred by the disease (see Section 2.1 for details). The SIR model classifies the population according to the stage of infection propagation and estimates the total number of infected people based on the changes in population between each group [6,7]. According to the classification of the population, the SIR model has several variants, such as the SEIR (Susceptible–Exposed–Infectious–Recovered) model [8], SLMCR (Susceptible–Latent–Mild/Critical–Removal) model [9], and SEIAQR (Susceptible–Exposed–Infected–Asymptomatic–Quarantined–Recovered) model [10]. 

Our study aimed to develop a modified SEIHR model to analyze the socioeconomic effect of the passenger reduction policy by using the COVID-19 infection risk estimation model developed by Park and Kim [4]. By analyzing multiple scenarios with different variables, we verified the applicability of the model and drew policy implications to prevent the spread of COVID-19 through public transportation.

The remainder of this study is organized as follows. Section 2 describes the method of calculating the social cost according to the implementation of the bus passenger reduction policy using the model of Park and Kim [4] and the modified SEIHR model. Section 3 demonstrates a practical application of the model. Section 4 discusses the results and key findings of the study. Finally, Section 5 presents the conclusion.

## 2. Materials and Methods

### 2.1. Optimal Number of Passengers to Minimize Total Social Cost

**Figure 1:**
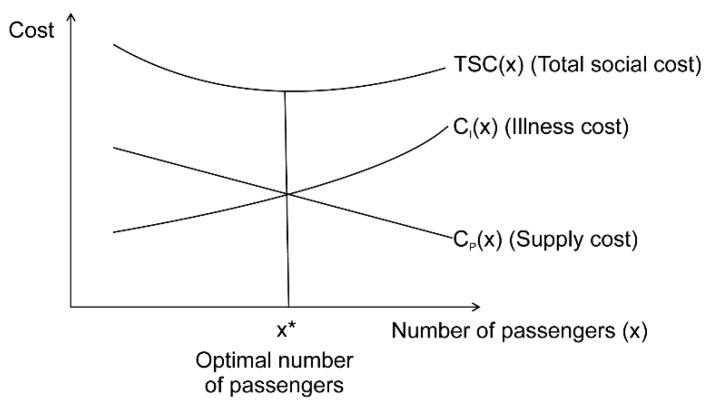
Optimal number of passengers (x*) to minimize the total social cost.

As the number of passengers per vehicle decreases, the probability of infection, and therefore, the total number of infected persons decreases. As the number of infected persons decreases, the cost of treatment for the infected and loss incurred due to restrictions on their economic activity decrease. Conversely, as the number of passengers per vehicle is reduced, the number of vehicles in operation must be increased to handle the current travel demand; accordingly, the cost of supplying public transportation services increases. Therefore, as the social illness cost decreases and the supply cost of the public transportation service increases owing to a reduction in the number of passengers, an optimal number of passengers per vehicle, which minimizes the total social cost, can be obtained by adding the two costs, as shown in Figure 1. The total social cost, which is the sum of the illness and supply costs, can be expressed as Equation (1).

(1) $$\mathrm{TSC}\left(x\right)=C_{I}\left(x\right)+C_{P}\left(x\right),$$

where TSC(x): total social cost, *C_I_*(*x*): illness cost, *C_P_*(*x*): supply cost, x: number of passengers in a vehicle.

### 2.2. Optimal Number of Passengers to Minimize Total Social Cost

Reducing the number of passengers on public transportation vehicles is effective in reducing the risk of infection. The various costs of illness can be reduced by reducing the probability of infection and the number of infected people. 

The social costs of managing the pandemic depend on the increase in the number of people infected with COVID-19. Medical facilities must be expanded, and the number of healthcare workers must be increased. Various medical expenses must be made to treat the infected people and conduct preemptive tests and epidemiological investigations. In addition to the direct investment costs, productivity losses caused by the inability of an infected person to engage in economic activities during the treatment period and operating losses due to reduced economic activity caused by social distancing policies can also be deemed as social costs.

Because it would be nearly impossible to accurately estimate all social costs, including direct investment costs and indirect costs, in this study, only the primary direct costs caused by new infections were considered, as shown in Equations (2)–(5). In other words, only medical expenses, epidemiological investigation expenses, and productivity losses during the treatment period of the infected patients were considered as the cost of illness. By assuming that these costs are simply proportional to the number of infected people, it was found that approximately 60 million KRW in socioeconomic costs per infected person was incurred (As of September 2022, 1 USD = 1395 KRW; 60 million KRW = 43,011 USD).

(2) $$C_{I}\left(x\right)=O\left(x\right)+V\left(x\right)+L\left(x\right),$$

(3) $$O\left(x\right)=n\left(x\right)\times \left(r_{m}\times c_{m}\times d_{m}+r_{s}\times c_{s}\times d_{s}\right),$$

(4) $$V\left(x\right)=n\left(x\right)\times \left(t\times c_{e}+t_{d}\times c_{q}\right),$$

(5) $$L\left(x\right)=n\left(x\right)\times \left(r_{a}\times d_{c}\times a+t_{d}\times r_{a}\times d_{q}\times a\right),$$

where *C_I_*(*x*): illness cost, *O*(*x*): healthcare cost, *V*(*x*): epidemiological investigation cost, *L*(*x*): cost of lost productivity, *n*(*x*): cumulative number of infected people during a unit period, *r_m_*: ratio of mild patients (0.9), *r_s_*: ratio of severe patients (0.1), *c_m_*: treatment cost for mild patients (KRW 220,000/day), *c_s_*: treatment cost for severe patients (KRW 650,000/day), *d_m_*: average number of treatment days for mild patients (24.5 days), *d_s_*: average number of treatment days for severe patients (21.5 days), *t*: average number of contacts (262 people), *t_d_*: average number of close contacts (25 people), *c_e_*: testing cost per capita (KRW 70,000), *c_q_*: self-isolation cost per capita (KRW 802,000), *r_a_*: ratio of the economically active population (0.637), *d_c_*: number of days of labor loss for confirmed cases (17.3 days), *d_q_*: number of days of labor loss for those under self-isolation (10 days), *a*: salary (KRW 94,000/day).

To estimate the effect of reduced infections through the policy to reduce the number of passengers using public transportation (Equation (2)), it is necessary to identify the number of infected people *n*(*x*) before and after the introduction of the policy. Park and Kim’s [4] model can estimate the reduction in the number of infected people on public transportation owing to the reduction in the number of passengers. However, public transportation users can be infected through other circumstances, and the effect of an infected person on public transportation can lead to secondary and tertiary effects on society as a whole. To estimate the effect of reducing the number of people on the number of infected people in society, it is necessary to consider the transmission of the disease in society as a whole. In other words, it is possible to estimate the effect of reducing the number of passengers in vehicles on the number of infected people by comparing the difference between the daily transmission of infection in society before and after implementing the passenger reduction policy. In this study, we propose a new model that combines the macroscopic infection transmission model, SEIHR model, and microscopic model of Park and Kim [4].

#### 2.2.1. Classical Compartmental Models in Epidemiology, SEIHR

The basic mathematical model for predicting the spread of infectious diseases is the SIR model [6,11]. This model classifies the population into the susceptible group (S)→infected group (I)→recovered group (R) according to the phase in the process until recovery and estimates the changes in the number of each cluster over time. Owing to the intuitiveness and extensibility of the SIR model, extended models have been proposed by various groups. This study focuses on Choi and Ki’s [12] SEIHR model.

As of 2021, under the COVID-19 quarantine system in South Korea, treatment is conducted under quarantine after an infection is confirmed. To efficiently reflect this system and prevent the spread of the disease, it is preferable to use the SEIHR model, which is an extension of the SIR model, rather than the SIR model. This model adds the exposure and quarantine phases to the SIR model to classify the population into the susceptible group (S)→exposed group (E)→infected group (I)→hospitalized group (H)→recovered group (R), and estimates the changes in the number of each cluster over time. The amount of change in each group during a unit period t of the SEIHR model is shown in Equations (6)–(11).

(6) $$\frac{dS}{dt}=-\beta \frac{I}{N}S,$$

(7) $$\frac{dE}{dt}=\beta \frac{I}{N}S-\kappa E,$$

(8) $$\frac{dI}{dt}=\kappa E-\alpha I,$$

(9) $$\frac{dH}{dt}=\alpha I-\gamma H,$$

(10) $$\frac{dR}{dt}=\gamma H,$$

(11) N=S+E+I+H+R,

where *N* denotes the total population, *S* denotes the number of people in the susceptible group, *E* denotes the number of people in the exposed group, *I* denotes the number of people in the infected group, *H* denotes the number of people in the quarantine group, *R* denotes the number of people in the recovered group, *t* denotes the unit period, *β* denotes the infection transmission rate, *κ* denotes the progress of the symptoms, *α* denotes the hospitalization rate, and *γ* denotes the recovery rate.

The SEIHR model is a macroscopic model that estimates the spread of infection in society by estimating the changes in the clusters over time. This model is suitable for examining the trend of the number of infected people from a macroscopic point of view or estimating the characteristics of a disease based on the data on the number of infected people. However, this model is limited in its ability to estimate the number of infected people according to the implementation of a new policy, such as the policy to reduce the number of passengers in transportation vehicles.

#### 2.2.2. Modified SEIHR Model

In the SEIHR model, the process by which the entire population, which is the susceptible group (S), is infected and converted into the exposed group (E) is determined by the infection transmission rate *β*, which may depend on the quarantine measures of the government. If *β* is reduced through the implementation of a policy to reduce the number of passengers on public transportation, the number of infected people who are converted from the susceptible group to the exposed group would be reduced. However, because *β* is the transmission rate obtained by considering both infections in public transportation vehicles and infections in daily life, it is not easy to estimate the change in *β* due to the implementation of the passenger reduction policy. Therefore, by linking the decrease in the number of infected persons due to the passenger reduction policy, which was estimated using Park and Kim’s [4] model, with the SEIHR model, the effect of the policy in terms of the spread of infection in the society can be reflected in the SEIHR model. The amount of change in each group during the unit period t of the modified SEIHR model is shown in Equations (12)–(17).

(12) $$\frac{dS\left(x\right)}{dt}=-\left[\beta \frac{I}{N}S-Iuq\left(N_{e}\left(X\right)-N_{e}\left(x\right)\right)\right],$$

(13) $$\frac{dE\left(x\right)}{dt}=\left[\beta \frac{I}{N}S-Iuq\left(N_{e}\left(X\right)-N_{e}\left(x\right)\right)\right]-\kappa E,$$

(14) $$\frac{dI}{dt}=\kappa E-\alpha I,$$

(15) $$\frac{dH}{dt}=\alpha I-\gamma H,$$

(16) $$\frac{dR}{dt}=\gamma H,$$

(17) N=S(x)+E(x)+I+H+R,

where u denotes the rate of use of public transportation, q denotes the frequency of using public transportation over a unit of time, *N_e_*(*X*) denotes the number of new infections per vehicle without implementing the policy, and *N_e_*(*x*) denotes the number of new infections per vehicle after implementing the policy.

*Iuq*(*N_e_*(*X*)−*N_e_*(*x*)) in Equations (12) and (13) is the part that has been modified to reflect the effect of the transportation policy implementation. This term indicates the decrease in the number of infected persons due to the transportation policy implementation. By including this term in Equations (12) and (13), it is possible to assess the number of infections occurring in a society in which the effect of the policy implementation is reflected.

The number of infected people, which decreases according to the implementation of the policy, *Iuq*(*N_e_*(*X*)−*N_e_*(*x*)), can be calculated by multiplying the number of times the infected people used the target public transportation mode during the analysis period, Iuq, by the decrease in the number of infections per vehicle after the policy implementation, (*N_e_*(*X*)−*N_e_*(*x*)). Here, *N_e_*(*X*) and *N_e_*(*x*) can be calculated using Park and Kim’s [4] estimation model. The basic formula for this model is as follows.

(18) $$N_{e}\left(x\right)=\frac{\left(x-m_{f}\right)m_{f}}{x\left(x-1\right)}\sum_{\forall i}\sum_{\forall j|i\neq j}(1-{\left(1-p\right)}^{n\left(i\leftarrow j\right)}),$$

where *x* denotes the number of passengers in a public transportation vehicle, *m_f_* denotes the number of infected passengers in the vehicle, *p* denotes the risk of infection due to a single virus, and *n*(*i*←*j*) denotes the number of viruses transferred from the jth passenger to the ith passenger.

Using the modified SEIHR model proposed thus far, it is possible to determine the overall infection transmission status in society during the analysis period, in which the effect of the policy to reduce the number of passengers in public transportation is reflected. By using Equation (19), it is possible to calculate the cumulative number of infected persons during the analysis period in Equations (3)–(5).

(19) n(x)=N−S(x),

where *S*(*x*) denotes the number of susceptible people at the end of the analysis.

#### 2.2.3. Parameters for Modified SEIHR Model

The parameters to be applied in the modified SEIHR model were set as follows: *α*, representing the quarantine rate per unit time, was set to 1/4, considering the average period of four days required until confirmation of the infection. *κ*, representing the manifestation of symptoms, was set to 1/4, assuming an average incubation period of four days. *γ*, representing the recovery rate per unit time, was set to 1/14 considering an average quarantine period of 14 days.

In addition, *β*, representing the infection transmission rate per unit time, was calculated by multiplying the basic reproduction number *R_0_* by α. However, in this study, the effective reproduction number *R_t_* was used instead of *R_0_*, which represents the basic characteristics of the disease, to calculate the infection transmission rate considering social distancing policies.

### 2.3. Supply Cost

#### 2.3.1. Estimation of Additional Supply Cost

Reducing the number of passengers on public transportation vehicles can reduce the number of infected people, thereby reducing the risk of infection as well as limiting the spread of the disease. However, to make this policy effective, additional vehicles must be supplied in proportion to the reduction in the number of passengers per vehicle. The supply cost also increases in proportion to the number of vehicles added.

If the number of passengers per vehicle on a public transportation route operating at an interval of *h* during time *T* is *X*, this route can satisfy the traffic demand $\frac{T}{h}\times X$ during the time *T*. If the number of passengers per vehicle changes to *x*, then the interval should be changed to *h’*. As $\frac{T}{h}\times X=\frac{T}{h^{\prime }}\times x$, $h^{\prime }=\frac{x}{X}\times h$. 

Further, when the round-trip travel time of this route is *t*, *N_V_* = *t*/*h* of vehicles is required to be operated at intervals of *h*. If the interval *h* is changed to *h’*, the number of required vehicles changes to *N_V_’* = *t*/*h’*. Therefore, $N_{V}{}^{\prime }=N_{V}\frac{h}{h\prime }$; here, if $h^{\prime }=\frac{x}{X}\times h$ is applied, $N_{V}{}^{\prime }=N_{V}\frac{X}{x}$.

To reduce the number of passengers per vehicle on public transportation routes, $N_{V}{}^{\prime }-N_{V}=N_{V}\left(\frac{X}{x}-1\right)$ additional vehicles are required. Therefore, the additional supply cost required to reduce the number of passengers from *X* to *x* is

(20) $$C_{P}\left(x\right)=\varphi \times \rho \times N_{V}\left(\frac{X}{x}-1\right),$$

where *φ* is the total number of routes, and *ρ* is the operating cost per vehicle.

#### 2.3.2. Additional Supply Cost to Increase Vehicle Operation

The additional supply cost required to implement a policy to reduce the number of passengers per vehicle on a public transportation route can be calculated by multiplying the operating cost per vehicle by the number of additional vehicles required. 

Table 1 shows the additional supply costs required to reduce the number of passengers on a 45-seater intercity bus in the metropolitan area of South Korea, where the average number of vehicles per route, $N_{V}$, is eight vehicles per day; the daily operating cost per vehicle, *ρ*, is KRW 740,000 and the total number of routes operated in the Seoul metropolitan area, *φ*, is 242. 

The capacity of an intercity bus in the Seoul metropolitan area is 45 passengers. Considering that up to 49 passengers are boarded during peak hours, the maximum number of passengers, *X*, was set to 49. The minimum number of passengers was set to 22, which was 50% of the capacity.

## 3. Results

### 3.1. Parameter Setting

Assuming that the travel demand is fixed, the additional supply cost of the bus is fixed according to the number of passengers in a vehicle. However, illness costs vary according to several factors. The problem is that the exact values of the variables are not known, or it is difficult to fix them at a single value because they change every moment. Therefore, this study aimed to calculate the optimal number of passengers per vehicle to ensure the social efficiency of the passenger reduction policy while changing the major variables affecting the illness cost within an appropriate range by applying the model developed in this study to intercity buses in the Seoul metropolitan area.

Park and Kim [4] estimated the risk of COVID-19 infection by setting the probability of infection by a single coronavirus, *p* = 0.0022 (0.22%). However, the delta and omicron mutant viruses discovered later were found to be much more contagious. Hence, in this study, *p* was set to one, three or five times the value used by Park and Kim [4].

Before the delta mutant of COVID-19 appeared, the daily number of people infected in the Seoul metropolitan area was <500. However, after the omicron mutant of COVID-19 appeared, more than 200,000 people were infected daily. Hence, the initial number of infected people, *I_0_*, was set to 1000–500,000.

In the modified SEIHR model, *β*, representing the infection transmission rate per unit time, was calculated by multiplying the basic reproduction number *R_0_* by *α*, which is the quarantine rate per unit time. *R_0_* was changed from 1.0 to 1.3 in increments of 0.5.

The illness cost for one infected patient was estimated to be approximately 60 million KRW using Equations (2)–(5). However, after the emergence of the omicron mutant of COVID-19, as the proportion of severe patients was lowered and the number of days for treatment and isolation was reduced, the illness cost per person became much lower than before. Hence, in this study, the illness cost per person was reduced by 0.1, 0.4, and 0.7 times.

The analysis period was set to a quarter (91 days), and it was assumed that the total population of the Seoul metropolitan area (*N*) was 26 million, the use rate of the intercity bus (*u*) was 10%, and the frequency of using the intercity bus per day (*q*) was two times.

### 3.2. Number of Passengers Minimizing the Total Social Cost

**Figure 2:**
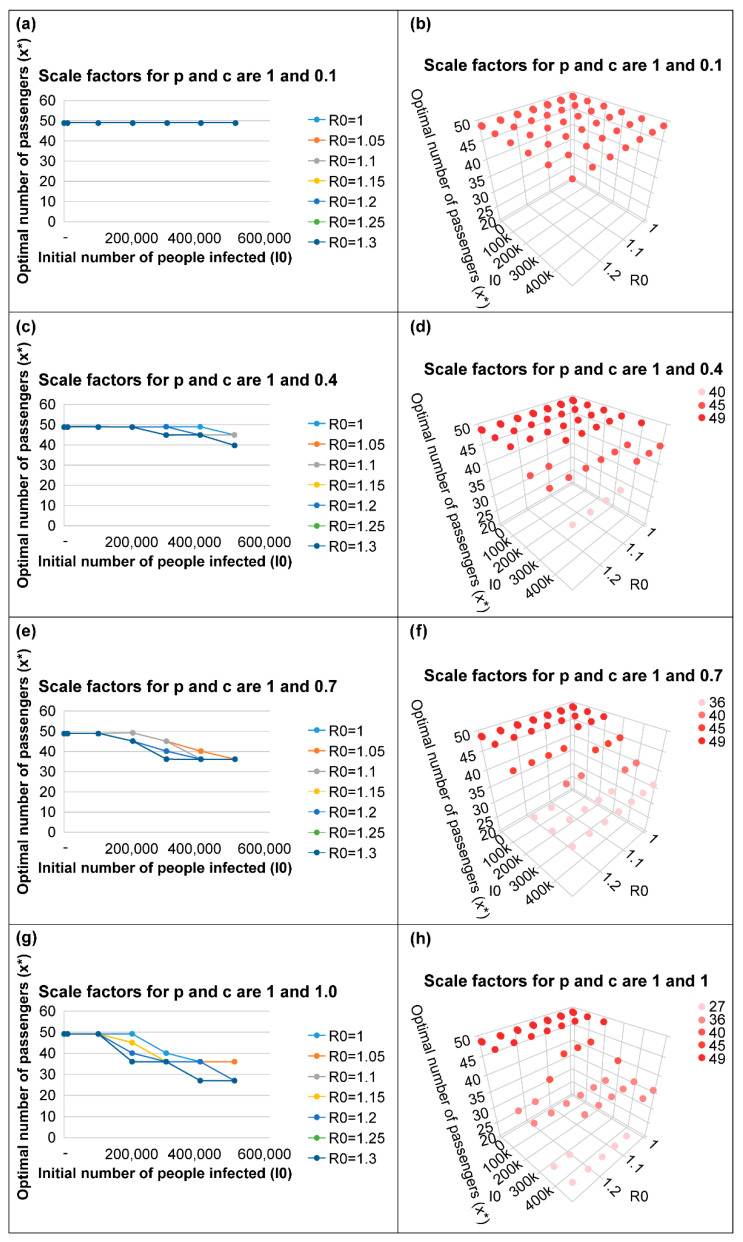
Optimal number of passengers to minimize total social cost when the scale factor for *p* is 1.

**Figure 3:**
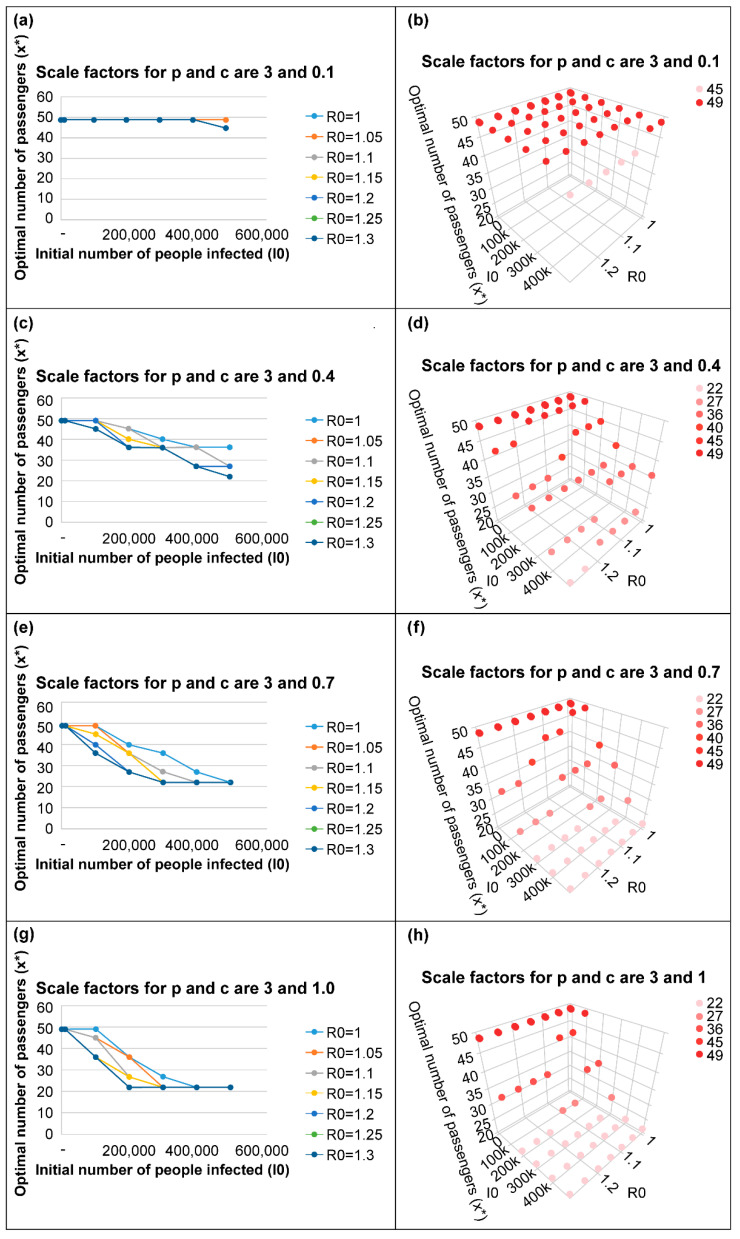
Optimal number of passengers to minimize total social cost when the scale factor for *p* is 3.

**Figure 4:**
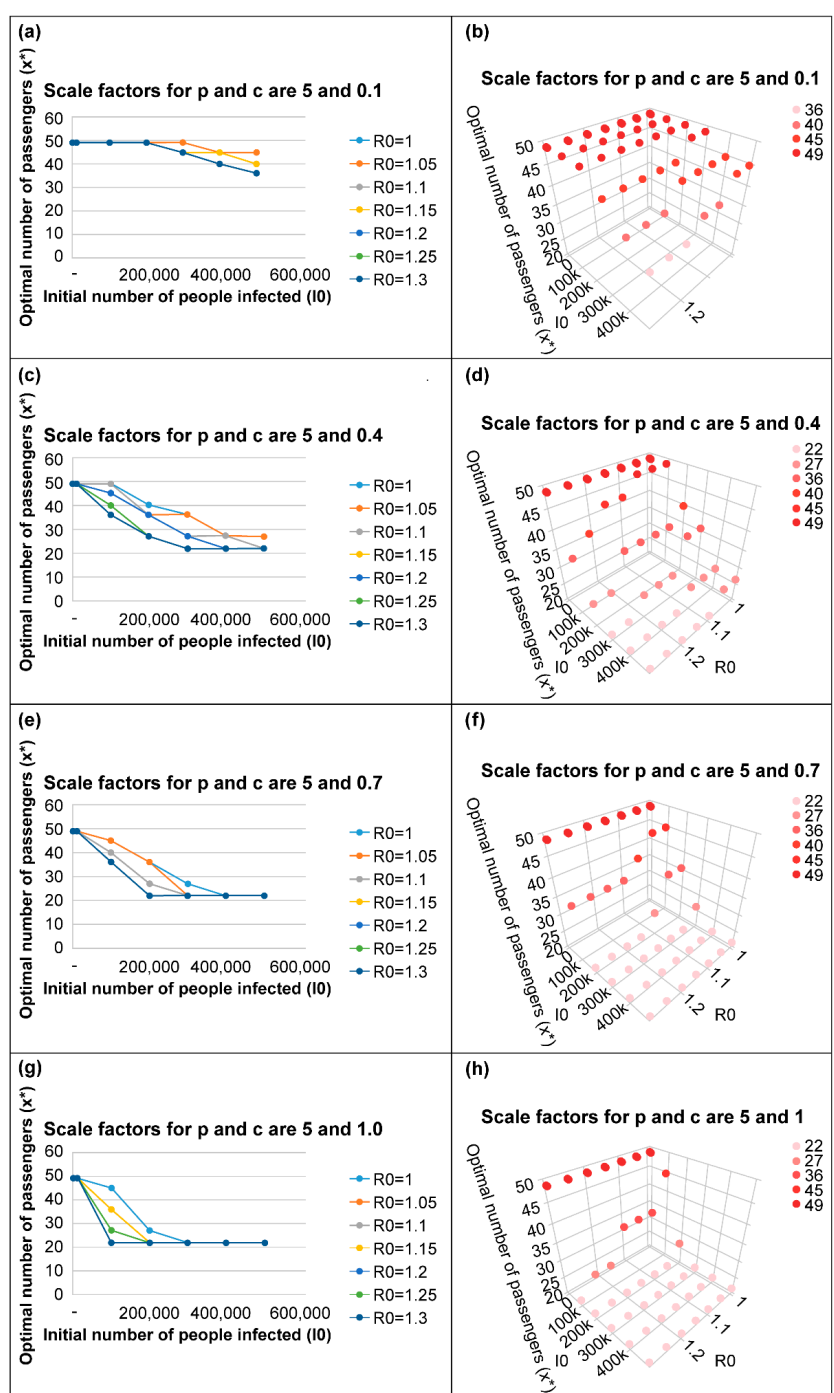
Optimal number of passengers to minimize total social cost when the scale factor for *p* is 5.

As shown in Figure 2, Figure 3 and Figure 4, as the number of new infections increases and the initial number of infected people increases, the policy of reducing the number of passengers per vehicle can minimize the total social cost.

The higher the basic reproduction number *R_0_*, the higher the spread of the disease; accordingly, reducing the number of passengers per vehicle minimizes the total social cost.

The higher the illness cost per person, the greater the reduction in the illness costs due to the reduction in the number of infected people; further, the higher the probability of infection by a single virus, *p*, the greater the number of infections; therefore, the policy of reducing the number of passengers per vehicle is more effective.

Figure 2c shows that in most cases, the optimal occupancy appears to be the initial value of 49 people; moreover, the optimal occupancy appears to be 45 or 40 when the initial number of infected people is very high and *R_0_* is large. In most cases, it can be seen that the policy to reduce the number of passengers per vehicle is not feasible. In this case, the scale factors for *p* and *c* are 1 and 0.4, respectively; therefore, this can be considered similar to the case where the probability of infection is approximately the same as that of original COVID-19 but the illness cost per person is very low, approximately the same as that of the omicron mutant of COVID-19. In other words, in the current situation, where the illness cost is low, the policy to reduce the number of passengers per vehicle will become ineffective if the probability of infection decreases in the future.

Figure 2g shows the situation where the scale factors for *p* and *c* are 1 and 1.0, respectively, which is similar to the initial COVID-19 situation in the Seoul metropolitan area. The policy to reduce the number of passengers per vehicle was found to be reasonable when the number of initially infected passengers exceeded 100,000. Considering that the daily number of infected people in the Seoul metropolitan area was approximately 500 in the initial stage of COVID-19, it can be seen that the policy to reduce the number of vehicles during that period was not feasible.

Figure 3e shows that the policy of reducing the number of passengers per vehicle appears reasonable when the initial number of infected passengers exceeds 100,000. In this situation, the scale factors for *p* and *c* are 3 and 0.7, respectively, which is similar to the situation corresponding to the delta mutant of COVID-19 in the Seoul metropolitan area. Considering that the number of infected people per day was tens of thousands at that time, a policy to reduce the number of passengers per vehicle could have been effective in reducing the total social cost.

As shown in Figure 4c, it appears that the policy to reduce the number of passengers per vehicle is reasonable when the initial number of infected people exceeds 1000. The situation where the scale factors for *p* and *c* are 5 and 0.4, respectively, can be considered similar to the situation in 2021 when the omicron mutant of COVID-19 emerged in the Seoul metropolitan area. In such a situation, it can be seen that the policy to reduce the number of passengers per vehicle can be an appropriate approach to reduce the total social cost.

However, Figure 4a shows that the passenger reduction policy was not effective for less than 200,000 passengers. Therefore, the situation where the scale factors for *p* and *c* are 5 and 0.1, respectively, is similar to the current situation in the Seoul metropolitan area. Hence, implementing a passenger reduction policy is currently considered unnecessary.

## 4. Discussion

The existing SIR series model was used to estimate the change in the number of infected people throughout the restricted region; however, it did not evaluate the effect of a specific policy. In this study, Park and Kim’s [4] model, which can estimate the spread of COVID-19 infection, was applied to the SIR series model, and a modified SEIHR model was developed to evaluate the effect of a public transportation policy to reduce the spread of COVID-19 infection. Using the developed model, we estimated the reduction in the illness cost due to the reduction in the number of passengers in public transportation and calculated the optimal number of passengers to minimize the total social cost in comparison with the increase in the vehicle supply cost.

While the cost of supplying a vehicle is fixed according to the number of passengers in the vehicle, the illness cost varies according to various variables. In this study, the results for various situations were derived by appropriately changing various variables and applying them to intercity buses in the Seoul metropolitan area.

The results showed that the optimal number of passengers decreased as the number of initially infected people increased; moreover, the basic reproduction number *R_0_*, illness cost per person, and probability of infection with a single virus, *p*, were higher. This result is consistent with common sense. Therefore, the model developed in this study is suitable for analyzing the socioeconomic effect of the COVID-19 response policy and can be applied to pandemic situations caused by other infectious diseases as well. However, depending on these variable conditions, the policy to reduce the number of passengers in a vehicle may not be required, so it is necessary to make an appropriate judgment according to the situation.

It was found that in the early stage of COVID-19 in Korea, a policy to reduce the number of passengers was not necessary because the number of infected people was not high (under 1000 people per day as of March 2021) owing to excellent countermeasures. However, when the number of infected people increased (around 3000 people per day as of March 2022) due to the delta and omicron mutants of COVID-19, a policy to reduce the number of passengers was found to be an appropriate approach to minimize the total social cost.

With the spread of the omicron mutant of COVID-19 appearing to be contained to some extent, the Korean government is planning to abolish the social distancing policy. As can be seen from the analysis results in this study, in the current situation where the illness cost is remarkably low, it is judged that the social distancing policy is no longer socioeconomically valid. Therefore, the Korean government is currently easing social distancing rules and striving for a return to normal life because the number of infected people is decreasing and the illness cost is significantly lower than that in the past.

## 5. Conclusions

The average number of passengers in intercity buses in the metropolitan area during peak hours is 49, which exceeds the maximum capacity of the vehicle. Because this is the average value calculated from the data of 200 routes, some routes have no standing passengers whereas some have more than 10 standing passengers. Although it is necessary to deploy additional vehicles to avoid standing passengers and ensure safety and convenience, it is also necessary to reduce the number of passengers per vehicle to lower the risk of COVID-19 infection, which also requires the additional deployment of vehicles. 

However, because an additional supply of vehicles increases the operating cost and lowers the operational efficiency, private bus carriers lack the incentive to adopt these measures on their own. To promote the policy to reduce the number of passengers on public transportation vehicles, the loss caused by the increased operating costs should be compensated by increasing the public transportation fare or providing financial support. Decisions regarding increasing the fare and providing financial support need to be made by the supervisory authority. Regardless of the selected approach, a policy to reduce the number of passengers on public transportation must be socioeconomically feasible. 

Given a situation where the COVID-19 pandemic continues for an extended period, we need to adapt to the situation and prepare to live our daily lives with COVID-19. In the past, there was considerable anxiety because of the lack of data and information regarding COVID-19; however, currently, people have become accustomed to the situation, and widespread vaccinations have mitigated the anxiety about COVID-19. Because the social and economic damage caused by COVID-19 is significant, efforts to reduce its spread need to be continued. Diverse policies should be proposed for public transportation, which has been a challenging area for implementing countermeasures. The effect of such policies can be maximized by analyzing the socioeconomic effect and implementing them at an appropriate time.

According to the results of this study, the social efficiency of the policy to reduce the number of passengers on intercity buses in the Seoul metropolitan area depends on the pandemic situation. The results of this study may vary depending on the variables. Therefore, the results can serve only as a reference for policy establishment. An appropriate analysis should be performed when implementing different policies under different circumstances. However, the significance of this study is that it presents a methodology to analyze the social efficiency of public transportation policies in response to COVID-19. The social efficiency analysis presented in this study can be applied to policies for responding to other infectious diseases worldwide.

## Figures and Tables

**Table 1:** Additional supply cost according to the extent of reduction in the number of passengers.

Passenger Reduction Level		Additional Vehicles and Supply Cost			
Crowdedness (Passengers /Capacity)	Passengers in a Vehicle x	Additional Vehicles Per Route (Veh/Route) $N_{V}{}^{\prime }-N_{V}=N_{V}\left(\frac{X}{x}-1\right)$	Additional Supply Cost Per Route (KRW Thousand Won/Route/Day) $\;\rho \times N_{V}\left(\frac{X}{x}-1\right)$	Total Number of Additional Vehicles (Vehicle) $\varphi \times N_{V}\left(\frac{X}{x}-1\right)$	Total Additional Supply Cost (KRW Million Won/Day) $C_{P}\left(x\right)$
-	(*X*=) 49	0	0	0	0
100%	45	0.7	526	172	127
90%	40	1.8	1332	436	323
80%	36	2.9	2138	699	517
70%	31	4.6	3437	1124	832
60%	27	6.5	4824	1577	1167
50%	22	9.8	7265	2376	1758

## Data Availability

Not applicable.
